# A Review of Soft Tissue and Nerve Masses in the Pediatric Hand: Pathology, Diagnosis, and Management Strategies

**DOI:** 10.7759/cureus.85999

**Published:** 2025-06-14

**Authors:** Zuhair Zaidi, Daniel Villarreal Acha, Amber McCranie, Jennifer Kargel

**Affiliations:** 1 Plastic Surgery, University of Texas Southwestern Medical School, Dallas, USA; 2 Plastic Surgery, University of Texas Southwestern Medical Center, Dallas, USA

**Keywords:** pediatric hand surgery, pediatric orthopedic surgery, peripheral nerve surgeries, plastic and reconstructive surgery, tumor, tumor surgery

## Abstract

Soft tissue and nerve masses of the pediatric hand, though often benign, prompt significant concern due to their visible location, potential for functional impact, and diagnostic uncertainty. Compared to adults, children demonstrate a distinct spectrum of hand lesions, with differences in prevalence, presentation, and pathology. Given the unique considerations in pediatric evaluation, including developmental anatomy and the importance of a child-appropriate approach to physical examination, clinicians must be equipped with a clear understanding of these lesions to guide appropriate management. This review synthesizes current literature on the pathology, diagnosis, and treatment of common pediatric hand masses, including cysts, lipomas, giant cell tumors, vascular tumors, and peripheral nerve lesions. By consolidating available evidence and expert recommendations, this article aims to support accurate clinical assessment and optimize care for pediatric patients presenting with soft tissue and nerve masses.

## Introduction and background

As a topic, pediatric hand masses remain relatively understudied within the field of musculoskeletal tumors. While the hands account for only 2% of the total body surface area, statistics indicate that more than 15% of all soft tissue tumors arise in the hands [1]. In the pediatric population, concerns about growing masses prompt families to seek urgent medical attention, posing unique challenges for clinicians due to the delicate nature of pediatric examinations. Additionally, the presentation and distribution of masses in pediatric hands can vary starkly from those in adults. While most tumors in the hand are benign with a favorable prognosis, malignant tumors are rare but feared occurrences that possess distinct characteristics and require astute care to prevent aggressive invasion or metastasis. Therefore, knowledge of tumor characteristics, specifically in pediatric patients, facilitates evaluation and diagnosis, improving overall outcomes. This review assesses the basic epidemiology, clinical presentation, imaging, and treatment options for soft tissue and nerve hand tumors and tumor-like lesions in the pediatric population.

## Review

Soft Tissue Masses

Ganglion Cysts

Ganglion cysts are the most common benign soft tissue tumors in the hand and wrist. They are characterized by a collagen wall connected to a joint or tendon sheath surrounding a thick mucin that may enter the space through a one-way valve [1-3]. A slight female predilection is observed, with a female-to-male ratio of approximately 1.4-2.7:1 [4]. Although common, the exact cause of ganglion cysts is not fully understood, but current theories implicate that repetitive microtrauma or capsular tears lead to mucinous degeneration of connective tissue [5]. Clinically, many pediatric ganglion cysts are asymptomatic and discovered incidentally, while others present as palpable lumps causing pain, tenderness, weakness, or cosmetic concerns [4]. Importantly, the distribution of ganglia in children varies by age: Volar wrist ganglions are predominant in patients younger than 10 years, whereas dorsal wrist ganglions are more frequently observed in older children and adolescents [6,7].

Dorsal Ganglion Cysts

Dorsal ganglion cysts usually arise from the scapholunate joint or ligament capsule [4,8,9]. On examination, a dorsal wrist ganglion presents as a smooth, soft, fluctuant swelling along the dorsal carpus. The cyst wall is firm but elastic, and its translucent, gelatinous contents often allow transillumination on penlight exams [4]. Diagnosis is primarily clinical; plain radiographs may be obtained to rule out bony pathology, and ultrasonography is a useful adjunct to confirm the cystic nature and detect occult ganglia, with a diagnostic accuracy of up to 87% [10]. MRI is reserved for atypical presentations or preoperative planning, as it can delineate the cyst and its stalk in relation to adjacent structures.

Many dorsal wrist ganglions in children are asymptomatic and can be observed; however, notably, these lesions have a lower likelihood of resolution compared to volar cysts [11]. When it becomes symptomatic (i.e., pain, discomfort with wrist extension, or limited range of motion) or if it enlarges, intervention is considered. Aspiration of a dorsal ganglion can be attempted, which is relatively safe in this location given the absence of major neurovascular structures, but simple needle aspiration in pediatric patients has a low success rate [12]. In a large pediatric series (654 wrist ganglions), nonsurgical measures like aspiration achieved resolution in only 18% of cases [13]. A newer minimally invasive option is percutaneous ultrasound-guided ganglion fenestration (PUGG), which involves aspiration and multiple fenestrations of the cyst wall under imaging guidance. Early results in children suggest PUGG can safely be performed without sedation and with no major complications, yielding resolution in the majority of cases [2]. A definitive treatment for persistent or symptomatic cysts is surgical excision. Open excision (or arthroscopic removal in select cases) includes resection of the cyst along with its stalk and a portion of the joint capsule to reduce recurrence. Surgical management in children has the highest cure rate (resolution in ~73% of cases in one study); however, even with meticulous excision, recurrence rates are higher in pediatric patients than in adults, reported anywhere from 6% to 35% [8,13,14].Notably, a history of prior aspiration can increase the difficulty of excision and has been associated with higher recurrence, presumably due to scarring and fragmented cyst remnants [15-17].

Volar Ganglion Cysts

Volar ganglion cysts occur on the palmar aspect of the wrist, often arising from the radiocarpal joint or adjacent intercarpal joints (such as the scaphotrapezoid or scaphotrapezial joints) [4]. Volar cysts typically present as palpable, sometimes tender masses on the wrist flexor side, most often radial, and share the same pathology as dorsal cysts, including a myxoid center, collagen wall, and joint-connected pedicle [15]. Volar ganglia require special consideration due to their anatomical location. Some may extend into the carpal tunnel, causing median nerve symptoms; however, more often, the concern is their close proximity to the radial artery - particularly at the radial-volar wrist. A thorough physical exam - including pulse assessment, along with transillumination, and, in some instances, ultrasound - is essential to confirm the cyst and rule out nearby involvement.

Management of pediatric volar cysts often starts with observation, as ~80% resolve spontaneously within two years, unlike dorsal cysts, which tend to persist [11]. If intervention is indicated - typically due to pain, functional impairment, or persistent concern - surgical excision is the definitive treatment for volar wrist ganglion cysts in children. It is indicated for symptomatic, enlarging, or refractory lesions and requires meticulous dissection to preserve the radial artery and adjacent nerves. Pediatric series report resolution rates of 70%-75% following excision, although recurrence remains higher in children than in adults [13,15]. Aspiration, while less invasive, is generally discouraged due to poor outcomes and anatomical risks. Specifically, recurrence rates exceed 80%, with Shanks et al. reporting resolution in only 18% of pediatric wrist ganglions treated with aspiration versus 73% with excision [13]. The risk of arterial injury is especially concerning for volar cysts, which often lie adjacent to the radial artery. The PUGG technique - a percutaneous, ultrasound-guided ganglion decompression - has been proposed as a safer aspiration alternative in anatomically sensitive cases and may be selectively considered in patients not amenable to surgery [2]. Nevertheless, the predominant approach remains conservative observation, with surgical excision reserved for cysts that are symptomatic, enlarging, or unresponsive to nonoperative management.

Giant Cell Tumor of the Tendon Sheath

Giant cell tumors of the tendon sheath (GCTTS) are benign soft tissue neoplasms arising from the synovial lining of the tendon sheath. GCTTS is the second most common tumor of the hand in adults with a peak incidence in the third to fifth decades and a slight female predominance. However, they rarely occur in children under the age of 10, with only a handful of cases published in the pediatric population [18]. The exact cause of GCTTS is unclear, but etiological factors proposed in adults may include metabolic disease, trauma, inflammation, and neoplastic origins [19]. GCTTS presents as a firm, non-fluctuant, nodular mass, commonly found on the volar aspects of the fingers and hands and is often slow-growing and painless [20].

Diagnosing GCTTS is challenging, as a combination of imaging and histological examination is typically necessary. Ultrasound is the preferred initial investigation, revealing a hypoechoic mass with insights into vascularity, size, number of satellite lesions, and tumor interactions [21-24]. Radiologically, GCTTS shows soft tissue shadowing, and recurrent cases display bony pressure erosions or intraosseous involvement [25,26]. MRI is valuable for diagnosing GCTTS, typically showing low signal intensity on T1- and T2-weighted images [27,28]. Fine needle aspiration cytology can assist in confirming GCTTS [29,30]. The histology of GCTTS is characterized by multinucleated giant cells, synovial cells, siderophages, foam cells, and inflammatory cells [31]. The tumors can present with varying colors and appearances, ranging from gray to yellow-orange with areas of brownish discoloration [32].

Marginal excision is the recommended treatment for GCTTS [33]. However, this method presents the added challenge of performing delicate surgery on very small anatomical structures in younger patients; therefore, precision in excision and avoiding violation of the capsule are critical [34]. Pediatric cases have shown no recurrence with this approach to date, however, few studies have long-term follow-up beyond one to two years [34].

For multiple recurrent or aggressive GCTTS lesions in adults, radiation therapy is considered to eliminate residual tumor cells post-surgery, although its use and effectiveness in the pediatric population are not yet well documented [23]. It is worth noting that a malignant form of GCTTS has been described, illustrating the potential for more aggressive behavior and emphasizing the importance of meticulous surgical techniques for the treatment of GCTTS without recurrence [35,36].

Epidermal Inclusion Cysts

Epidermal inclusion cysts, also known as epidermoid cysts or epidermal cysts, are common benign cutaneous lesions of the hand [37,38]. They can develop from trauma or occlusion of pilosebaceous follicles, resulting in the introduction of epithelial cells into the dermis and the subsequent formation of a keratin-filled cyst [39]. There is some clinical variation; however, an epidermal inclusion cyst typically appears as a fluid-filled bulge just deep into the skin that communicates with the surface via a punctum [37]. While most are benign, larger cysts have a rare chance of malignancy [40].

Histologically, they are characterized by a stratified squamous epithelial lining with central lamellated keratin debris [41]. Imaging with ultrasound or MRI can aid in delineating borders and assessing for deep extension, though preoperative MRI is not routinely indicated unless malignancy or involvement of adjacent structures is suspected [42]. Although not initially necessary, biopsies are often helpful to rule out malignancy in cases where the mass appears more aggressive on exam or radiography.

Surgical excision is the preferred management for symptomatic or enlarging cysts. Complete resection with preservation of the cyst capsule is essential to minimize recurrence. Although specific pediatric data are limited, adult studies suggest that violating the cyst wall can lead to keratin spillage, foreign body reaction, and recurrence rates up to 3%-5% [43]. Extrapolating from adult literature, surgeons should prioritize maintaining capsule integrity during pediatric excision until more targeted studies are available.

Lipomas

Hand lipomas are benign masses that represent just 4% of soft tissue tumors in children [44-46]. They originate from mesenchymal primordial fatty tissue cells and are characterized by slow growth, a soft texture, and a lack of tenderness [47]. These mobile masses typically have a "doughy" consistency and do not transilluminate like ganglion cysts [48]. Lipomas near peripheral nerves can cause compression neuropathy, leading to symptoms like paresthesia and a positive Tinel test [49].

Soft tissue biopsy in conjunction with diagnostic imaging techniques such as radiography, ultrasound, and MRI is used to diagnose lipomas [50]. On plain radiographs, lipomas may demonstrate the “Bufalini sign,” which appears as well-defined radiolucent areas that are more transparent than adjacent muscle, helping to distinguish them from other soft tissue masses [51]. MRI is particularly valuable in evaluating these tumors, typically revealing a well-defined, homogeneous mass with high signal intensity on both T1- and T2-weighted images, mirroring the signal of subcutaneous fat [52]. The signal attenuates on fat-suppressed sequences, confirming the fatty composition. The absence of thick septa, nodular components, or contrast enhancement helps distinguish benign lipomas from liposarcomas.

In pediatric patients, asymptomatic and stable hand lipomas may be managed conservatively with observation. Surgical excision may be performed to prevent complications, especially in cases where functional and neurological symptoms are present. Liposarcomas should be ruled out in cases of giant lipomas, especially if they demonstrate rapid growth, exceed 5 cm, or are present intramuscularly [53,54]. Radiological images help assess the probability of malignancy, in which case complete excision is performed. Recurrence rates following excisional biopsy or marginal excision are generally low in the adult literature but are not well documented in the pediatric population. Although uncommon, vigilance for malignant degeneration should be maintained.

Glomus Tumors

Glomus tumors are rare vascular growths developing from the glomus body, a neuromyoarterial structure located below the nail, which helps regulate blood pressure and temperature in the skin [55]. These tumors occur more commonly in females and typically appear as painful, oval-shaped, blue or red subungual nodules sensitive to temperature [56,57]. They are often idiopathic but may also develop following an injury or secondary to neurofibromatosis [57].

Diagnosing glomus tumors involves recognizing a triad of symptoms that differentiate them from other masses such as neuromas. This triad includes local sensitivity, severe pain with pressure, and cold hypersensitivity, with the last being pathognomonic for a glomus tumor [58,59]. Other diagnostic tests, such as the Love pin and Hildreth, are also used to confirm the diagnosis. The Love pin test involves applying pressure to the suspected area, with the point of severe pain indicating the tumor's location. The Hildreth test induces transient ischemia using a tourniquet, with a positive result indicated by pain relief in the affected area. Although children with glomus tumors often present early due to pain and tenderness, the mass may be too small to detect on the exam and may take several years to present with the triad of symptoms. For this reason, while not always necessary, imaging - especially MRI, which can detect tumors as small as 2 mm - helps confirm diagnosis and rule out mimics like venous malformations or schwannomas [60,61]. X-rays may additionally reveal bony erosion caused by the tumor.

The prognosis of glomus tumors in children is generally favorable with complete surgical excision. Two approaches, the lateral subperiosteal and the transungual approach, are used for excision, each with its own considerations [62]. The lateral subperiosteal approach, while limited in the visual field and less effective for centrally located tumors, can preserve the nail unit and minimize nail deformities. The transungual approach offers a broader visual field and better exposure for central tumors but may result in deformities due to nail bed damage [62]. The choice of approach depends on factors like tumor location, vascular supply, and the importance of preserving nail aesthetics. In cases of recurrence or malignant transformation, further evaluation and resection are necessary. Malignant glomus tumors are rare in children but can recur locally and occasionally present as multifocal lesions [63]. Given their rarity in children, literature on pediatric cases is limited, but evidence supports complete excision and vigilant follow-up to manage these tumors.

**Figure 1:**
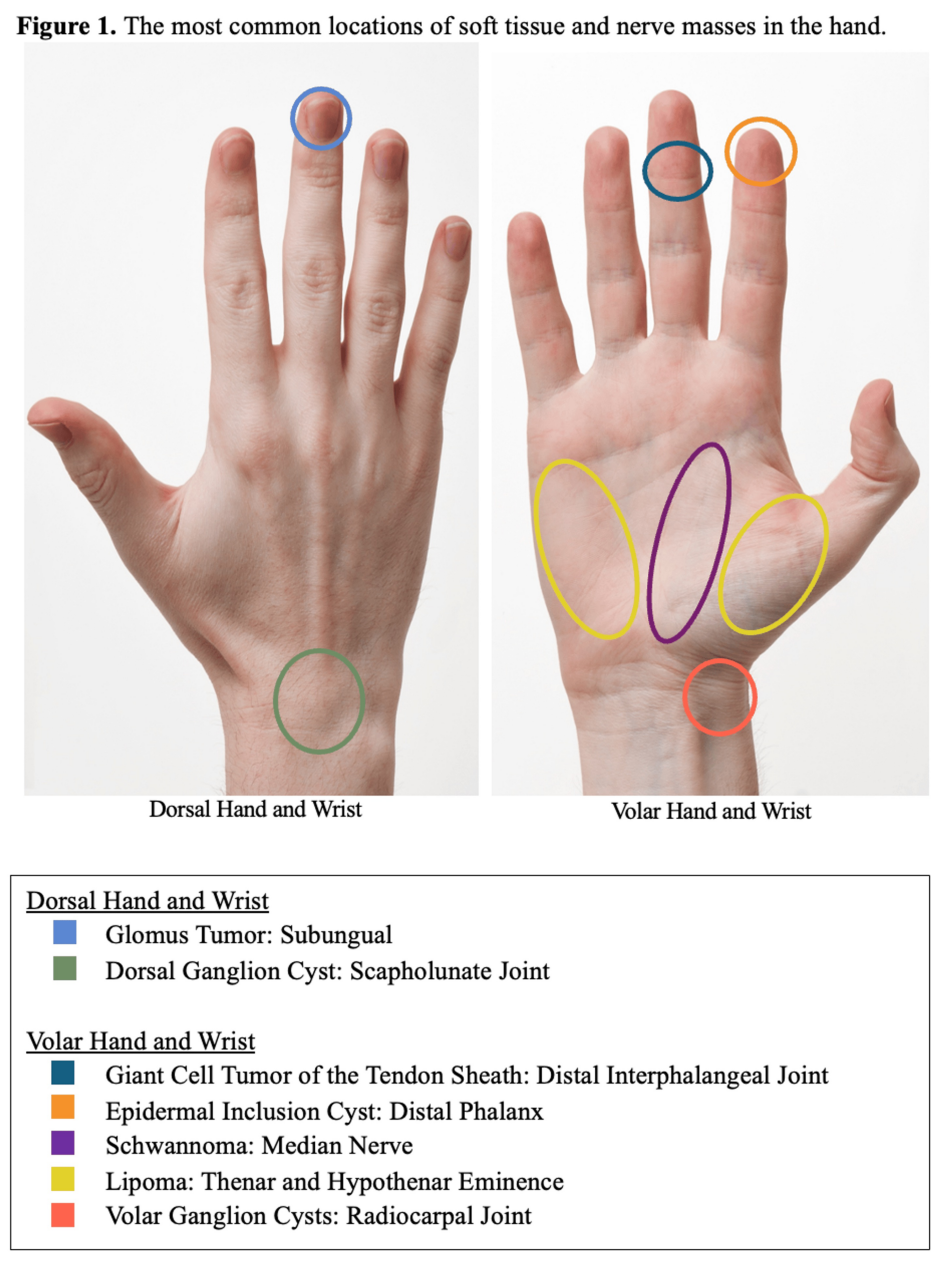
The most common locations of soft tissue and nerve masses in the hand Dorsal view (left) and volar view (right) illustrate typical sites for glomus tumors (blue) [59], dorsal ganglion cysts (green) [1], giant cell tumors of the tendon sheath (dark blue) [20], epidermal inclusion cysts (orange) [37], schwannomas (purple) [92], lipomas (yellow) [52], and volar ganglion cysts (red) [1]. Not pictured: Infantile hemangiomas commonly appear in a “biker-glove” distribution or on distal digits of either the dorsal or volar hand [68]. Neuromas typically occur at sites of nerve injury or amputation [87]. Source: “Human-Hands-Front-Back” by Evan-Amos, licensed under CC BY-SA 3.0 (https://commons.wikimedia.org/wiki/File:Human-Hands-Front-Back.jpg).

The most common locations of soft tissue and nerve masses in the hand are shown in Figure 1.

Hemangioma

Hemangiomas are benign pediatric hand tumors caused by abnormal proliferation of endothelial and vascular architecture [64,65]. Hemangiomas expand gradually, presenting as painless lesions with a soft-rubbery texture upon palpation [66]. Superficial hemangiomas are bright red lobulated papules or plaques, while deeper ones may be blue or skin-colored [66]. Throbbing pain may arise, particularly when the hand is positioned below the heart due to venous engorgement [65,66].

Infantile hemangiomas (IHs) are the most common benign vascular tumors in infants, affecting 4%-5% of newborns [64]. They are more prevalent in Caucasian infants, with a female-to-male ratio of up to 5:1. Risk factors include prematurity, low birth weight, and advanced maternal age. While IHs occur in the head and neck region, they may present in a biker-glove distribution on the hand and distal digits [67,68]. IHs emerge within the first few weeks of life, proliferate rapidly during the initial months, and begin involution around 12 months, often resolving by the age of 4-7 years [67,69]. Most IHs regress on their own, but some may require intervention to control growth and address cosmetic concerns. The reported incidence of IHs varies, ranging from 1% to 3% among infants and up to 22% to 30% in preterm infants [70].

Congenital hemangiomas (CHs), in contrast, are fully formed at birth and subdivided into rapidly involuting (RICH), non-involuting (NICH), and partially involuting (PICH) types [71]. CHs often appear as purple or red nodules with coarse telangiectasias and a pale rim, commonly overlying joints or digits. RICHs begin involution soon after birth and typically resolve by 12-14 months, while NICHs persist and may require resection if symptomatic. Upon imaging with MRI, IHs may be differentiated by more well-circumscribed borders and different T1- and T2-weighted signal intensities when compared to CHs [67]. 

Treatment for hemangiomas depends on the type and severity of the lesion. Conservative management including observation may be sufficient for some cases, while others require surgical removal. In the case of IHs, oral propranolol is the first-line treatment [72]. Topical timolol and laser therapy are options for superficial lesions or residual skin changes. Other medications like corticosteroids, vincristine, and interferon-α have been used but may cause associated complications [70,73]. Vascular malformations can be treated with sclerotherapy, which is performed by injecting sclerosants directly into the malformed cavities, damaging the endothelium [74,75]. Surgical resection may be indicated if sclerotherapy is not feasible or unsuccessful.

Recurrence of hemangiomas is rare, especially in those that achieve complete regression [76]. However, residual fibrofatty tissue or other complications may require additional treatment. Long-term follow-up is important to monitor the progression and recurrence of these vascular tumors.

Nerve masses

Neuromas

Pediatric hand neuromas are diagnosed based on clinical evidence and physical examination [77]. They develop as a result of nerve injury and are characterized by disorganized bundles of microfascicles created by misdirected axonal regeneration [78,79]. Symptoms include persistent pain, sensory disturbances, or the presence of a tender mass or scar [80,81]. While research on painful neuromas in children is limited, a positive Tinel sign, pain localized to a specific nerve distribution, and the use of local anesthetic injections may aid in diagnosing neuromas [82-84]. Conservative approaches like massage and desensitization are initially recommended for treatment, but upon failure to alleviate symptoms, surgical intervention is necessary. Surgery involves excising the neuroma and providing an appropriate pathway for axonal regrowth via nerve grafting or transposition [85]. Alternative approaches include transcutaneous electrical stimulation, topical lidocaine, nerve blockades, and Botox injections for pain management [85-87].

Schwannomas

Schwannomas are the most common solitary peripheral nerve tumors in the pediatric hand, accounting for less than 5% of soft tissue neoplasms [88]. The exact cause of schwannomas is uncertain, and the current literature hypothesizes repetitive trauma or foreign bodies as potential stimulants for the growth of Schwann cells [89,90].

Schwannomas appear as painless, slow-growing masses in individuals aged 20-50 years, with a higher incidence in females [91]. They are commonly found on the volar surfaces of the hand and wrist. These tumors originate from Schwann sheath cells and possess distinct features, including mobility within the nerve, a well-encapsulated and pedunculated appearance, and no infiltration into nerve fascicles [92].

Diagnosing schwannomas may be challenging, as they can be mistaken for other soft tissue masses. Ultrasonography and MRI aid in the diagnosis by revealing hypoechoic, homogeneous masses with posterior acoustic enhancement [92]. However, intraoperative biopsy is often necessary to definitively differentiate schwannomas.

The treatment for schwannomas involves intracapsular removal under magnification to preserve neurological function [92,93]. Surgery should involve careful removal of the tumor from the affected nerve. The tumor should be shelled out without disturbing any fascicles. In cases of large and long-standing schwannomas in the distal digital area, debulking or nerve reconstruction may be considered after resection.

Malignant transformation in schwannomas is rare, and the recurrence rates after surgical excision are low. However, there have been reported cases of recurrence in different areas of the nerves within the same extremity, possibly due to incomplete tumor removal or misdiagnosis of multiple tumors as solitary [93]. Therefore, careful monitoring and accurate diagnosis are crucial for the effective management of schwannomas.

## Conclusions

Pediatric soft tissue and nerve tumors of the hand are most often benign, with ganglion cysts representing the most frequently encountered lesion. Other commonly seen masses include hemangiomas, giant cell tumors, epidermal inclusion cysts, neuromas, and schwannomas, each presenting with unique clinical features, imaging characteristics, and treatment considerations. Accurate diagnosis relies on a thorough history, physical examination, and appropriate imaging, while management should be tailored based on symptom burden, lesion behavior, and potential for functional impairment. This review consolidates current evidence and clinical experience to provide a structured framework for evaluating and managing these tumors, equipping hand surgeons and pediatric providers with practical insights to ensure timely and effective care.
